# A cross-sectional study to determine the seroprevalence of bluetongue virus serotype 8 in sheep and goats in 2006 and 2007 in the Netherlands

**DOI:** 10.1186/1746-6148-4-33

**Published:** 2008-08-27

**Authors:** Armin RW Elbers, Johan Popma, Sandra Oosterwolde, Piet A van Rijn, Piet Vellema, Eugène MA van Rooij

**Affiliations:** 1 Department of Virology, Central Veterinary Institute (CVI) of Wageningen UR, PO Box 65, 8200 AB, Lelystad, The Netherlands; 2 Department of Small Ruminant Health, Animal Health Service, PO Box 9, 7400 AA Deventer, The Netherlands

## Abstract

**Background:**

In August 2006 a major epidemic of bluetongue virus serotype 8 (BTV8) started off in North-West Europe. In the course of 2007 it became evident that BTV8 had survived the winter in North-West Europe, re-emerged and spread exponentially. Recently, the European Union decided to start vaccination against BTV8. In order to improve the understanding of the epidemiological situation, it was necessary to execute a cross-sectional serological study at the end of the BT vector season. Cattle were the target species for cross-sectional serological studies in Europe at the end of 2006 and 2007. However, there was no information on the BTV8-seroprevalence in sheep and goats.

**Results:**

On the basis of our cross-sectional study, the estimated seroprevalence of BTV8-exposed locations in the Netherlands in 2006 was 0% for goats (95% confidence interval: 0 – 5.6%) and 7.0% for sheep (95% confidence interval: 3.5 – 12.9%). The estimated seroprevalence of BTV-8 exposed locations in 2007 was 47% for goats (95% confidence interval: 36 – 58%) and 70% for sheep (95% confidence interval: 63 – 76%). There was a wide range in within-location seroprevalence in locations with goats and sheep (1 – 100%). A gradient in seroprevalence was seen, with the highest level of seroprevalence in the southern Netherlands, the area where the epidemic started in 2006, and a decreasing seroprevalence when going in a northern direction.

**Conclusion:**

There is a much higher estimated seroprevalence of locations with goats exposed to BTV8 than can be inferred from the rather low number of reported clinical outbreaks in goats. This is probably due to the fact that clinical signs in infected goats are far less obvious than in sheep. The wide range in within-location seroprevalence observed means that the proportion of animals protected in 2008 by a natural infection in 2006 and/or 2007 can differ highly between flocks. This should be taken into account when vaccinating animals.

## Background

In August 2006 a major epidemic of bluetongue virus serotype 8 (BTV8) started off in North-West Europe, including the Netherlands, Belgium, Germany, Luxembourg and the North of France [1,2]. In order to improve the understanding of the epidemiological situation of this disease, it was necessary to execute a cross-sectional serological study at the end of the vector season of 2006. The Community legal framework on bluetongue monitoring and surveillance was laid down in Council Directive 2000/75/EC and Commission Decision 2005/393/EC and these are in line with the Terrestrial Animal Health Code of the OIE. Cattle were the target species for the cross-sectional serological study at the end of 2006 [3,4].

Many hoped that the winter season of 2006/2007 would halt the BTV epidemic, assuming that the chain of transmission would be broken by the dying off of infected adult vectors and a halt in the life cycle of the vector because of low temperatures. However, in the course of 2007 it became evident that BTV8 somehow had survived the winter in North-West Europe and a re-emerging epidemic spread exponentially within the original affected countries. Moreover, BTV8 was introduced into the United Kingdom, Denmark, Czech Republic and Switzerland [5].

The scale of the epidemic in 2007 was so huge that the European Union decided to start vaccination against BTV8 in 2008. The vaccination campaign aims to achieve a 80% or more coverage of animals protected (either by vaccination or by immunity acquired through natural infection) [6].

The sentinel monitoring system, set up at the beginning of 2007 to detect re-emergence of BTV8, already provided some insights into the extend of the BTV8 spread in the cattle population in 2007. However, with respect to goats and sheep there was no information on the BTV8-seroprevalence in North-West Europe. This paper presents the seroprevalence and geographical spread of BTV8 on animal and herd levels in goats and sheep in the Netherlands in 2006 and 2007.

## Methods

Blood samples from Dutch sheep and goats were serologically tested at the Central Veterinary Institute (CVI) in Lelystad for antibodies against BTV8 using a competitive ELISA (Institute Pourquier, Montpellier, France). This ELISA has a high sensitivity (~100%) and specificity (>99.8%) [7]. The blood samples were collected in the framework of obligatory and voluntary health programmes (e.g. certified disease-free programmes within the European Union) executed by the Dutch Animal Health Service. For the 2006 seroprevalence estimates, we used blood samples collected in the first months of 2007. For the 2007 seroprevalence estimates, we used blood samples collected in the last months of 2007. Locations with animal sampled were selected proportional to the population distribution of locations with goats and sheep in the Netherlands in the different provinces. Sample size within locations for the health programmes was set at detecting at least a prevalence of disease of 5%. In smaller flocks this often meant that almost all animals within the flock were sampled.

There are approximately 41,000 locations with sheep and 23,000 locations with goats in the Netherlands registered at the Animal Health Service. Based on these data and an a priori estimated seroprevalence of BTV8-infected locations with goats of 5% and a maximum acceptable error in the estimated prevalence of 5% and a 95% confidence level required for the estimated prevalence, a sample size of approximately 70 locations with goats was calculated using WinEpiscope version 2.0 [7] for our cross-sectional study in 2006. For 2007, we adjusted the a priori estimated seroprevalence of BTV-8 infected locations with goats to 30% with a maximum acceptable error in the estimated prevalence of 10% and a 95% confidence level required for the estimated prevalence, resulting in a sample size of approximately 81 locations with goats for 2007. For the locations with sheep, we used an a priori estimated prevalence of BTV8-infected locations with sheep of 10% and a maximum acceptable error in the estimated prevalence of 5%, resulting in a calculated sample size of approximately 140 locations with sheep for 2006. For 2007, we adjusted the a priori estimated seroprevalence to 50% with a maximum acceptable error in the estimated prevalence of 7.5% and a 95% confidence level required for the estimated prevalence, resulting in a sample size of approximately 170 locations. Since there is also an unknown number (not registered) of small-holder locations in the Netherlands with goats and sheep with a small numbers of animals (1 to 5 animals per location), we arbitrarily increased the actual sample size with approximately 15 to 20%. Exact confidence intervals for the estimated seroprevalence were calculated according to Fleiss [9].

## Results

### Seroprevalence of BTV8-infected locations with goats and sheep in 2006

**Figure 1:**
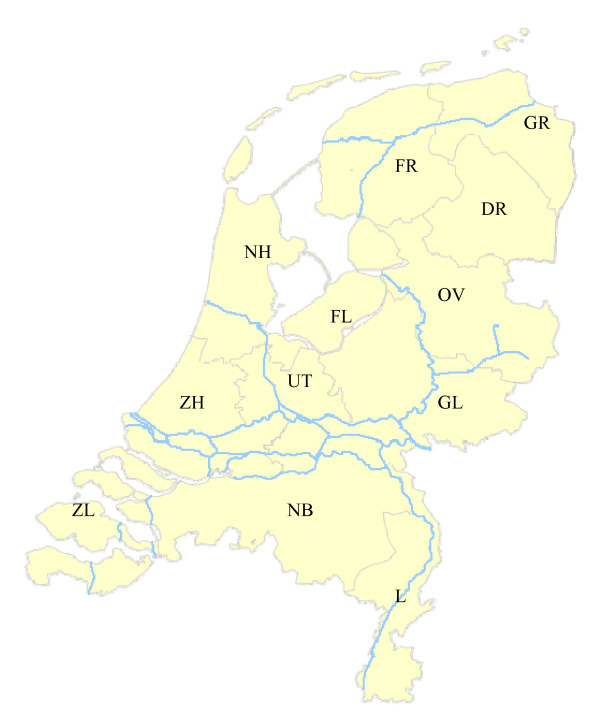
**Geographical location of the twelve provinces in the Netherlands** (DR: Drente; FL: Flevoland; FR: Friesland; GL: Gelderland; GR: Groningen; L: Limburg; NB: North Brabant; NH: North Holland; OV: Overijssel; UT: Utrecht; ZH: South Holland; ZL: Zeeland).

A total of 1,975 goat sera from 83 locations with goats and 2,555 sheep sera from 143 locations with sheep were collected. All goat samples were seronegative, a total of 17 sheep samples (0.7%) from 10 locations with sheep (7.0%) were seropositive. The BTV8-seropositve locations with sheep were located in the provinces of Limburg, North Brabant, Zeeland, and Gelderland. Within-location seroprevalence ranged from 3 to 50% (almost all sheep present in the flocks were sampled). The location of the provinces in the Netherlands is shown in Figure 1. Based on our sampled population we estimated the proportion of BTV8-seropositive locations in the Netherlands with sheep and goats. On a national level the estimated seroprevalence of BTV8-exposed locations in 2006 was 0% for goats (95% confidence interval: 0 – 5.6%) and 7.0% for sheep (95% confidence interval: 3.5 – 12.9%).

### Seroprevalence of BTV8-infected locations with goats and sheep in 2007

A total of 1,995 goat sera from 81 locations with goats and 4,252 sheep sera from 214 locations with sheep were collected. A total of 204 goat samples (10.2%) from 38 locations with goats (46.9%) were seropositive. The BTV8-seropositve locations with goats in 2007 were located in the provinces of North Holland, South Holland, Utrecht, Gelderland, North Brabant, and Limburg.

**Figure 2:**
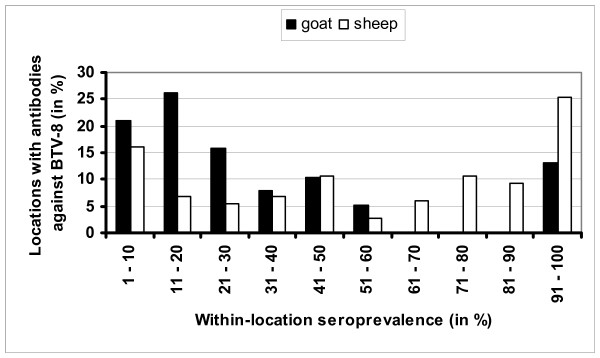
Distribution of within-location seroprevalence on locations with antibodies against bluetongue virus serotype 8 in goats (N = 38) and sheep (N = 149) in the Netherlands in 2007.

A total of 1,627 sheep samples (38.3%) from 149 locations with sheep (69.6%) were seropositive. The BTV8-seropositve locations with sheep were located in all provinces of the Netherlands in 2007. On a national level the estimated seroprevalence of BTV8-exposed locations in 2007 was 47% for goats (95% confidence interval: 36 – 58%) and 70% for sheep (95% confidence interval: 63 – 76%). The median within-location seroprevalence on BTV8-infected locations with goats was 21% (min – max: 1 – 100%) and with sheep 67% (min – max: 1 – 100%) (Figure 2).

## Discussion

There is very sparse information on seroprevalence of BTV-infected locations with goats and sheep during outbreaks. A cross-sectional study in Kazakhstan showed a within-herd seroprevalence in cattle, sheep and goats varying between 0 and 100% [10]. A cross-sectional study in sheep flocks in Northern Pakistan [11] indicated 90% of the flocks seropositive. In these seropositive sheep flocks, within-flock seroprevalence ranged from 12 to 100% (median: 47%). A cross-sectional serological study in Queensland, Australia, found within-flock seroprevalences in infected sheep flocks ranging from 1 – 42% and in infected goat flocks ranging from 5 – 16% [12].

During the 2006-epidemic in North-West Europe, no clinically affected goats were reported [3]. The results of our seroprevalence study is in line with those findings. However, in week 35 of 2007, the first clinical disease in goats caused by BTV8 in North-West Europe was reported from the Netherlands [13]: in a holding containing 600 milking goats, 10 goats demonstrated clinical signs of BT, starting with acute drop in milk yield and pyrexia, followed by edema of lips and face, crusts on lips and muzzle, nasal discharge, conjunctivitis and erythema of the udder. Up to the end of 2007, a total of 25 holdings reported clinical disease (BT indicative) in goats in the Netherlands. The results of our seroprevalence study indicate a seroprevalence of locations with goats of approximately 50% in the Netherlands in 2007. This is much higher than can be inferred from the 25 locations that reported clinical outbreaks in goats in 2007. This is probably due to the fact that clinical signs in infected goats are far less obvious than in sheep [14].

In the first year (2006) of the BTV8-epidemic in the Netherlands, a total of 270 sheep flocks and 200 cattle herds reported clinical disease. In 2007, the epidemic really took off: about 3,200 clinical outbreaks were reported by locations with sheep on a total of 6,500 outbreaks reported. The results of the seroprevalence study in sheep in 2006 and 2007 are in line with the number of clinical outbreaks reported.

In our study we see a wide range in within-location seroprevalence in locations with goats and sheep. This means that the proportion of animals protected in 2008 by a natural infection in 2006 and/or 2007 can differ highly between locations. Two options for a vaccination strategy are open: either one vaccinates all ruminants on the locations with animals irrespective of the proportion of animals protected by a natural infection in 2006 or 2007 or one determines the proportion of animals protected by a natural infection within the flock: if a low to moderate proportion of animals is naturally protected one has a good reason to vaccinate all the animals; if a high proportion of animals is naturally protected, this might be a reason not to vaccinate the flock.

## Conclusion

Seroprevalence of BTV8-exposed locations with goats and locations with sheep was much higher in 2007 than in 2006. There was a much higher estimated seroprevalence of locations with goats exposed to BTV8 than can be inferred from the rather low number of reported clinical outbreaks in goats. This is probably due to the fact that clinical signs in infected goats are far less obvious than in sheep. There was a wide range in within-location seroprevalence in locations with goats and sheep. This means that the proportion of animals protected in 2008 by a natural infection in 2006 and/or 2007 can differ highly between flocks. This should be taken into account when vaccinating animals.

## Authors' contributions

ARWE, PAvR and EMAvR designed the study. PV facilitated the use of sera collected by the Animal Health Service. JP and SO performed the laboratory analyses. ARWE performed the data analyses and drafted the manuscript. JP, PAvR, PV and EMAvR commented on the draft. All authors read the manuscript and approved the manuscript.
